# Population Pharmacokinetic Method to Predict Within-Subject Variability Using Single-Period Clinical Data

**DOI:** 10.3390/ph14020114

**Published:** 2021-02-03

**Authors:** Won-ho Kang, Jae-yeon Lee, Jung-woo Chae, Kyeong-Ryoon Lee, In-hwan Baek, Min-Soo Kim, Hyun-moon Back, Sangkeun Jung, Craig Shaffer, Rada Savic, Hwi-yeol Yun

**Affiliations:** 1 College of Pharmacy, Chungnam National University, Deajeon 34134, Korea; fjdwh01@gmail.com (W.-h.K.); tianods89@gmail.com (J.-y.L.); jwchae@cnu.ac.kr (J.-w.C.); 2 Division of Convergence Technology New Drug Development Center, Osong Medical Innovation Foundation, Cheongju 28160, Korea; 3 Laboratory Animal Resource Center, Korea Research Institute of Bioscience and Biotechnology, Ochang 28116, Korea; kyeongrlee@kribb.re.kr; 4 College of Pharmacy, Kyungsung University, Busan 48434, Korea; baek@ks.ac.kr; 5 College of Pharmacy, Pusan National University, Busan 46241, Korea; minsookim@pusan.ac.kr; 6 Department, Pharmaceutics, Ernest Mario School of Pharmacy, Rutgers, The State University of New Jersey, New Brunswick, NJ 08854, USA; hmback89@gmail.com; 7 Department of Computer Science and Engineering, Chungnam National University, Daejeon 34134, Korea; hugmanskj@gmail.com; 8 Department of Bioengineering and Therapeutic Sciences, School of Pharmacy, University of California, San Francisco, CA 94158, USA; craig.shaffer@ucsf.edu

**Keywords:** interindividual variability, residual variability, pharmacokinetics, statistical modeling

## Abstract

Sample sizes for single-period clinical trials, including pharmacokinetic studies, are statistically determined by within-subject variability (WSV). However, it is difficult to determine WSV without replicate-designed clinical trial data, and statisticians typically estimate optimal sample sizes using total variability, not WSV. We have developed an efficient population-based method to predict WSV accurately with single-period clinical trial data and demonstrate method performance with eperisone. We simulated 1000 virtual pharmacokinetic clinical trial datasets based on single-period and dense sampling studies, with various study sizes and levels of WSV and interindividual variabilities (IIVs). The estimated residual variability (RV) resulting from population pharmacokinetic methods were compared with WSV values. In addition, 3 × 3 bioequivalence results of eperisone were used to evaluate method performance with a real clinical dataset. With WSV of 40% or less, regardless of IIV magnitude, RV was well approximated by WSV for sample sizes greater than 18 subjects. RV was underestimated at WSV of 50% or greater, even with datasets having low IIV and numerous subjects. Using the eperisone dataset, RV was 44% to 48%, close to the true value of 50%. In conclusion, the estimated RV accurately predicted WSV in single-period studies, validating this method for sample size estimation in clinical trials.

## 1. Introduction

Target sample sizes in clinical trials are calculated based on various components, including type I error, type II error, significance level, power, and variability. Variability, especially within-subject variability (WSV), is the most important factor to determine sample size. Although the other components can be controlled through clinical trial design, variability is dictated by subject characteristics. The variability from study subjects can be separated into interindividual variability (IIV) and WSV. However, since at least two periods of data are necessary to calculate the exact WSV, statisticians may use total variability, not WSV, when calculating sample sizes. The use of total variability may overestimate the sample sizes for clinical trials and cause ethical or economic issues due to inefficient clinical trial design [1,2,3,4,5,6]. The ethical issues related to determining the number of subjects are particularly critical. For a long time, a small number of subjects was considered an unethical approach due to their low scientific value. However, more recently, it has been reported that an unethical and inadequate number of subjects is being decided on to cover statistical risk in clinical trials, and an appropriate and ethical approach was suggested, namely, size of effect (delta, Δ), which is a critical factor determining the number of subjects [7].

A population-based method is a statistical method using mixed effects to reflect population characteristics. At the population level, variability can be expressed as a combination of fixed and random effects. Fixed effects are represented by structural parameters and covariate effects, whereas random effects are characterized by variability, such as interindividual variability (IIV) and residual variability (RV). Population-based methods have been used to explain pharmacokinetic (PK) studies because they enable the distinction between variability from within or between subjects [5,6,8]. Nonlinear mixed effect modeling (NONMEM) is typically used to analyze fixed and random effects simultaneously in PK results. In NONMEM, the fixed effects (theta (*θ*) of NONMEM) can be expressed by a single value or with equations to define relationships between parameters, such as the definition of PK parameters alone or relations between covariates and PK parameters. Random effects represent the distributional variance element of the model. NONMEM random effect estimates consider IIV (eta (*η*)) to have a mean of zero and variance of omega squared (*ω^2^*), whereas RV (epsilon (ε)) has a mean of zero and variance of sigma squared (*σ^2^*). Theoretically, RV originates from the difference between the true and observed values that result from interassay variability, model misspecification, and process noise [8,9,10]. However, RV from these sources can be minimized by validation of the bioanalytical method and quality controls for clinical trials based on guidelines of good clinical practice. Thus, most RV can be approximated as the within-subject variability (WSV) in well-designed and controlled clinical trials. Regardless of difficulty for WSV quantitation, high WSV could generate problems in clinical studies, such as the decision of subject number or difficulty with consistency for suitable statistical power. Especially in bioequivalence studies, highly variable drugs that exhibit area under the plasma concentration–time curve (*AUC_t_*) and maximum plasma concentration (*C_max_*) values with WSV >30% could have difficulty in deriving equivalence, even though the FDA and EMA have expanded the bioequivalent limit in their guidelines for bioequivalence studies on highly variable drugs [11,12,13,14,15,16].

We hypothesize that WSV may be predicted accurately and efficiently using single-period clinical trial results. The purpose of this study is to explore the performance of a population-based method to accurately predict WSV with simulated single-period clinical trials, validate this method by applying it to a measured clinical dataset collected after administration of eperisone (known as a high WSV drug), and determine whether this method could be used to improve sample size prediction in clinical trials.

## 2. Results

### 2.1. Performance of a Population-Based Method Using a Virtual Single-Period Clinical PK Trial

**Figure 1:**
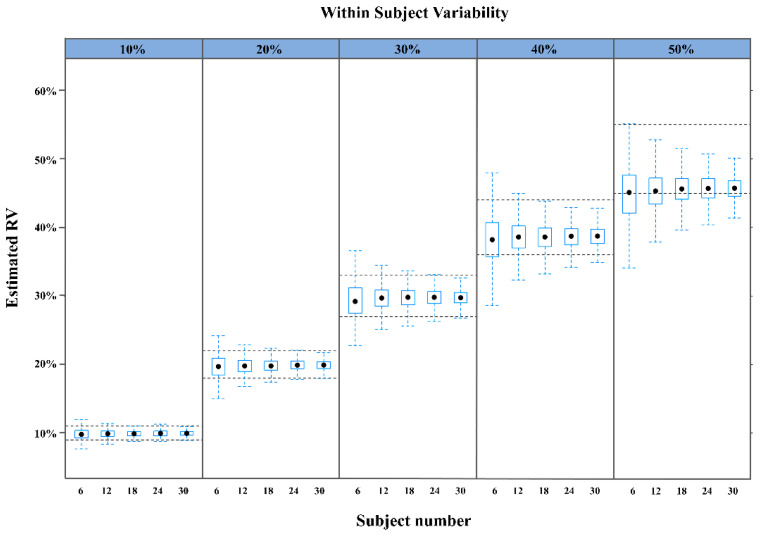
Residual variability (RV) in the first experiment. Approximation of RV for various levels of within-subject variability (WSV) and numbers of study subjects using the datasets of the first experiment.

The first experiment used a simulated dataset generated by R (Text S1: example of R code for generating simulation dataset), which contained various WSV levels but unchanged IIV. The estimated RVs closely approximated the true values when WSV varied from 10% to 40%. At WSV of 50%, the generated RV underestimated the true value (Figure 1, Table 1). The proportion of predictive success was >90% when WSV was 10% to 30% for ≥12 subjects and when the WSV was 40% for ≥18 subjects (Figure S1). At WSV of 50%, the proportion of predictive success for all numbers of subjects were <66%.

**Figure 2:**
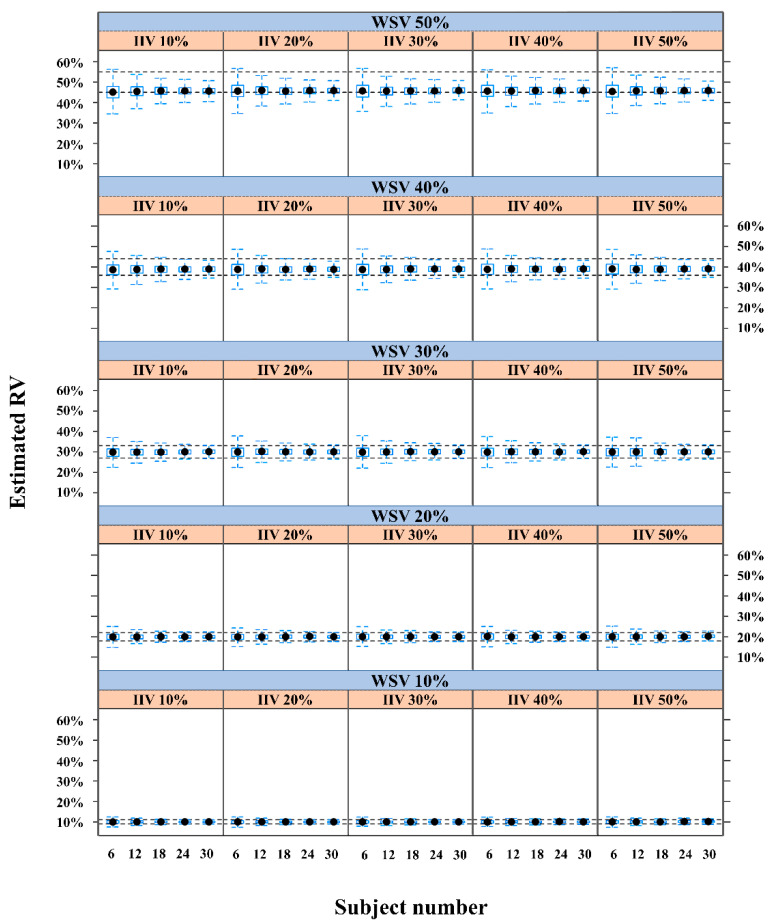
Residual variability (RV) in the second experiment. Approximation of RV for various levels of within-subject variability (WSV), interindividual variability (IIV), and numbers of study subjects using the datasets of the second experiment.

In the second experiment, the proportion of predictive success was >90%, regardless of IIV value, when WSV varied from 10% to 40% for ≥18 subjects (Table 2, Figure 2 and Figure S2). The proportion of predictive success was <68% when WSV was 50% at all IIVs, even with the largest number of study subjects (30 subjects).

The proportion of predictive success was above >90% at WSV from 10% to 40% for >18 subjects, regardless of IIV level; however, predictive success was low at WSV 50%. In addition, we explored the covariance effect between IIVs of clearance (*CL*) and volume of distribution (*V_d_*) to evaluate the correlation effect between them; however, there were no significant differences with or without covariance (Table S1, Text S2). As the data simulations assumed no interaction between IIV, *CL,* and *V_d_*, the results did not incorporate covariances into the predictions. 

### 2.2. Real Case Application

WSV (CV_w_, coefficient of within-subject variation) for *C_max_* (maximum drug concentration) of eperisone (dose, 50 mg) was 50.21%, as reported previously [11], suggesting that eperisone is a highly variable drug. To evaluate the present population-level method with a clinical dataset, random sampling for the generation of a single-period dataset was performed. For various numbers of subjects randomly sampled from PK data with a total of 33 subjects, the RVs ranged from 44% to 48% (Table 3). Therefore, the estimated RVs were similar to the real WSV despite the use of a small sample size.

## 3. Discussion

The main objectives of this study were to verify how well population approaches could estimate RV compared with WSV in a single-period PK clinical trial, which simulated datasets under various conditions. In general, IIV and RV stand for eta (*η*) and epsilon (*ε*) in population approaches; it can cause a separation of variabilities with random effects. Basically, unexplained RV can result from experimental error, assay variability, model misspecification, and process noise. However, well-designed clinical trials, including PK studies, incorporate bioanalytical method validation; assay variability is a minor contributor to RV. In addition, clinical studies, including bioequivalence, typically follow good clinical practices, so other errors (e.g., bioanalytical errors) except WSV are minimized in RV. Therefore, WSV is the principal component of RV. Hence, WSV can be predicted as the entire RV in population-based methods; the epsilon (RV) was used to judge how close the estimated RVs approximated the WSVs. 

According to results from datasets consisting of IIV equal to zero, RV estimation was affected by sample size and magnitude of WSV size. The increase in sample size definitely tended towards the fact that the population approach was able to predict an RV close to theoretical WSV size. It was a predictable result since the degree of freedom would be larger than a low sample size, and variability would be smaller, consequently. In addition, RV could be well estimated up to around 40% of WSV using population approaches. If the single-period clinical trial was performed with a sample size of over 18 subjects and total variability was calculated to be under 40%, population approaches could be closely predicted for WSV because total variability had to be smaller than WSV. However, the interesting point was not to reach the 90% proportion of predictive success in all sample sizes; the profile looked like a saturable curve (Figure S1). That meant that population approaches were not only poor predictors of WSV, they would also not be improved by increasing the sample size to over 50% WSV. Therefore, for clinical trial results with over 50% total variability, population approaches can be carefully accessed to predict WSV.

The interaction between IIV and WSV was considered in the prediction of RV. The magnitude of WSV had a more dominant effect than IIV when predicting RV (Figure S2). Regardless of the IIV level, the predictive power was sufficient at WSVs of 10% to 40%, when the sample had >18 subjects. However, predictive power was insufficient for a WSV of 50%, possibly because the population-based method could not estimate IIV accurately with large unexplained RV. Similar to the results of 50% WSV without IIV in the first experiment, the dataset consisting of 50% WSV, with various IIVs, could not predict the RV as an alternative to WSV using a population-based method; the variability could not be clearly separated between IIV and WSV in that case.

Typically, the IIV and RV are treated as different parameters in population-based methods. Therefore, sufficient information for each variability parameter is needed for accurate predictions of IIV and RV. A sufficient sample size is necessary to accurately define IIV because IIV originates from interindividual error. In the current study, a sample of 6 subjects was too small to describe IIV because the predictive power was about 70% in all conditions. In cases where preclinical and clinical studies are performed with 6 subjects in a single-period study, results may be inaccurate. 

Although we evaluated the interaction between IIVs (not IIV and WSV) to determine the covariance effect between IIV and structural parameters, there were no significant differences with or without covariance (Table S1). The covariance of IIVs between structural parameters (*CL* and *V*) was not involved in the PK modeling because the covariance was not reflected when we generated simulated datasets. 

In the real case application, with data from 3 × 3 bioequivalence clinical trials (33 subjects), the within-subject coefficient of variance (*CV_w_*) for eperisone was *AUC* 33% and *C_max_* 50% [11]. To test the performance of population-based methods for the prediction of WSV in real clinical trials, the clinical trial dataset was used to perform a random sampling of 6, 12, 18, 24, and 30 subjects. The observed range of *C_max_* of RVs from randomly sampled datasets (44% to 48%) for 6 to 30 subjects was an underestimate; however, the range was sufficiently close to the real value of 50%. The difference in *AUC* (33% vs. 44–48%) may have been related to the calculation methods of RV. When we obtained RV from the population-based method, we used the −2-log likelihood to choose the best fit in a range of plasma concentrations. Since the −2-log likelihood was calculated using the sum of likelihood based on differences between the prediction and observation of each dependent variable, it would be more accurate to calculate RV with *C_max_* than *AUC* because the method for *AUC* is close to integral, not likelihood.

In fact, WSV estimation by population approach could be assessable for all drugs; however, it would be a more informative method for highly variable drugs (e.g., simvastatin, verapamil, nadolol, and propafenone [17,18,19,20]) than the others through the estimation of reliable WSV. In addition, our approach would be helpful to establish a reasonable and cost-reducible strategy for clinical or bioequivalence studies. The pros of the method were the fact what it could not only design clinical study efficiently but could also be expanded to covariate analysis in population PK analysis. The additional minimization of WSV could be expected to connect with covariate analysis. Nevertheless, the main cons of the method were that the clinician’s empirical decision was still required for sample size adjustment in the case of clinical trials of new drug candidates that do not have any information on human PK, variability, or covariates.

There may be a possible limitation of this study. The result for the applied example of our population method was obtained only for one highly variable drug (eperisone). Therefore, it will be necessary to evaluate the population method approach for other drugs as a further study. In addition, the researchers have to be aware that our method was confirmed based on a single-period study, so it needs to be implemented carefully when WSV estimation over a two-period study is performed.

## 4. Materials and Methods

### 4.1. Overall Scheme to Evaluate Performance of the Population-Based Method

**Figure 3:**
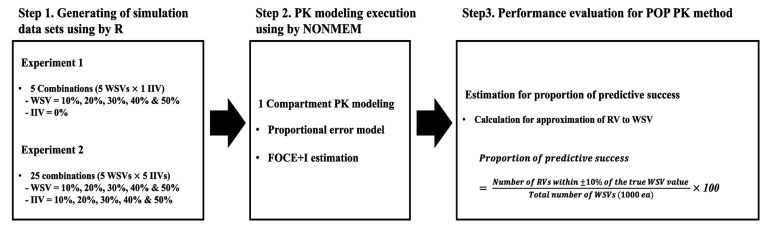
Experimental scheme. In Step 1, common simulation conditions for Experiment 1 and Experiment 2 were as follows: dose, 100 mg; CL, 10 L/h; V_d_, 50 L; sampling point, 12 points (from 0 to 24 h); subject number, 6, 12, 18, 24 and 30. Abbreviations: RV, residual variability; IIV, interindividual variability; WSV, within-subject variability; CL, clearance; V_d_, volume of distribution; FOCE+I, first-order conditional estimation with interaction; POP PK, population pharmacokinetics.

To test the theoretical validity of IIV and WSV values, a single-period virtual clinical PK trial was simulated with various combinations of IIVs and WSVs. The structural model, explained with clearance (*CL*) and volume of distribution (*V_d_*) as parameters, was assumed to be a 1-compartment model, with intravenous administration of the virtual drug. Simulated datasets were generated using statistical software (R, R Studio, and NONMEM) for the population-based method to estimate RV (NONMEM sample code provided in Text S3). For the first experiment, 1000 datasets were simulated using 5 different WSVs without IIVs. The dataset, consisting of combinations between 5 different IIVs and WSVs, was created for additional experiments (Figure 3). 

The mean population value of *CL* was set at 10 L/h and *V_d_* at 50 L, and the variance of structural parameters and plasma concentrations were set based on values of IIV and RV. The exponential error was used to describe IIV, and the proportional error was used to describe RV. Both errors were assumed to have a mean equal to zero and standard deviation equal to the square root mean of the desired value (Text S1 is sample R code; see supplement material). The chosen values *CL* and *V_d_* were based on general PK characteristics of drugs when taken as medicine once or twice daily. *CL*, *V_d_* and half-life values were assumed by 10 L/h, 50 L, and 3.5 h, respectively. In general, the half-life (2–5 h) could be understood to be typical of an oral drug taken once or twice a day, and the chosen values were also confirmed with literature to verify that the values were real [21,22,23,24,25].

### 4.2. Generating a Simulation Dataset and Performing Population PK Modeling for Scenarios without Changes in IIV (First Experiment)

In the first experiment, the simulation dataset was generated (R) with intravenous dosing; dose 100 mg; blood sampling times 0, 0.083, 0.167, 0.333, 0.5, 1, 2, 4, 6, 8, 12, and 24 h; *CL* 10 L/h (IIV, 0%); volume of distribution (*V_d_*) 50 L (IIV, 0); WSV 10%, 20%, 30%, 40%, and 50%; 6, 12, 18, 24, and 30 study subjects. As the conditions for 5 different study sizes were tested for each of the 5 levels of WSV, there were 25 total scenarios, with 1000 datasets for each scenario (Table 4). Data simulation was conducted (NONMEM) for population PK modeling using a 1-compartment intravenous model with a proportional error model. The estimation method was a first-order conditional estimation with an interaction option. The sigma values resulting from each scenario were collected as estimated RVs.

### 4.3. Generating Simulation Datasets and Performing Population PK Modeling for Scenarios with Differing WSVs and IIVs (Second Experiment)

In the second experiment, the simulation dataset was generated (R) with intravenous dosing; dose 100 mg; blood sampling times 0, 0.083, 0.167, 0.333, 0.5, 1, 2, 4, 6, 8, 12, and 24 h; *CL* 10 L/h (IIV 10%, 20%, 30%, 40%, and 50%); *V_d_* 50 L (IIV 10%, 20%, 30%, 40%, and 50%); WSV 10%, 20%, 30%, 40%, and 50%; 6, 12, 18, 24, and 30 study subjects. It was assumed that the IIVs of CL and V_d_ changed equally. As 5 levels of IIV were assigned to each level of WSV and 5 different numbers of subjects were allocated for each of 25 combinations (5 IIVs × 5 WSVs), the total number of scenarios was 125, and there were 1000 datasets for each scenario (Table 4). The PK modeling (NONMEM) and method for verifying the predictive power of PK modeling were identical to those in the first experiment.

### 4.4. Evaluation of Predictive Power

The RVs estimated (NONMEM) were evaluated at 80% confidence, within ±10% of the true WSV. The proportion of predictive success was calculated as the number of acceptable RVs, where it falls in the above criteria, divided by the total number (1000 ea) of RVs for percent description.

### 4.5. Application to Clinical Example (Real Case)

Bioequivalence studies for determining WSV were obtained (Korea United Pharmaceutical Co. Ltd., Jeondong, Sejong, Korea). The reference drug was eperisone 50 mg (Murex, Cho Dang Pharm Co., Ltd., Seoul, Korea), which is a high variability drug according to various related guidelines and publications [11,12,13,14,15]. The CV_w_ value, corresponding to WSV of *C_max_*, was 50.21%, as reported previously [11]. We randomly sampled groups of 6, 12, 18, 24, and 30 study subjects from real clinical data of eperisone and performed PK modeling using a 1-compartment model. Predictive power was compared with the same method used to evaluate predictive power for the randomly sampled single-period datasets. 

### 4.6. Softwares

Statistical software (R v. 3.6.1; R Studio v. 1.2.1335; Microsoft Excel 2016) was used to generate the simulation datasets to describe single-period PK clinical trials and analyze the results from the population-level method. Additional software was used to determine the variability terms (IIV and RV) using population-level methods (NONMEM v. 7.4.0; Pirana v. 2.9.8; PsN v. 4.9.0).

## 5. Conclusions

In summary, when we conducted population PK modeling with virtual single-period PK datasets of various scenarios, the estimated RV closely predicted WSV. When the WSV value was set below 50%, regardless of the magnitude of IIV, RV was approximated by WSV for 18 or more subjects, with high predictive success (90%). Application of the population PK method to randomly extracted single-period clinical PK data from a clinical 3 × 3 bioequivalence test confirmed method accuracy. Therefore, population-based methods may efficiently and accurately predict WSV in single-period clinical trials and improve sample size estimation.

## Figures and Tables

**Table 1:** Proportion of predictive success for various numbers of study subjects and levels of within-subject variability with no interindividual variability (first experiment).

WSV (%)	Proportion of Predictive Success (%) * for Each Subject Number				
	*n* = 6	*n* = 12	*n* = 18	*n* = 24	*n* = 30
10	75	91	95	99	99
20	75	91	96	98	99
30	71	90	96	98	99
40	66	84	90	94	96
50	51	53	62	63	66

* Proportion of predictive success, at which estimated sigma (RVs) values are included in the true value, with WSV of ±10%. Abbreviation: WSV, within-subject variability.

**Table 2:** Proportion of predictive success for various numbers of study subjects and levels of within-subject variability and interindividual variability (second experiment).

Setting Condition		Proportion of Predictive Success (%)* for Each Subject Number				
WSV (%)	IIV (%)	*n* = 6	*n* = 12	*n* = 18	*n* = 24	*n* = 30
10	10	72	85	94	97	99
	20	68	87	94	98	99
	30	73	86	94	96	97
	40	69	87	90	92	93
	50	70	82	88	88	85
20	10	70	86	93	96	98
	20	74	87	94	97	99
	30	71	87	94	96	98
	40	70	88	95	95	99
	50	72	86	87	97	98
30	10	73	88	93	98	99
	20	70	86	92	96	98
	30	68	88	92	96	98
	40	69	84	94	96	98
	50	71	72	91	96	97
40	10	71	82	89	94	98
	20	66	83	90	95	96
	30	70	84	92	94	96
	40	70	87	91	94	97
	50	71	85	90	94	97
50	10	50	56	62	62	63
	20	54	62	59	66	65
	30	55	58	61	64	68
	40	56	59	65	65	67
	50	54	60	62	66	68

* Proportion of predictive success at which estimated sigma (RVs) values are included in the true value, with WSV ±10%. Abbreviation: WSV, within-subject variability; IIV, interindividual variability

**Table 3:** Residual variability for single-period and measured clinical datasets.

Single Period Dataset *		Measured Dataset [11]
No. of Subjects	RV (%)	CV_w_ for C_max_ (%)
6	45	50.21
12	48	
18	45	
24	44	
30	47	

* Single-period trial, 33 subjects; no. of subjects sampled randomly. Datasets were made using data for individual pharmacokinetic data points of the reference drug, as previously published [11]. Abbreviations: C_max_, maximum concentration; CV_w_, coefficient of within-subject variation; RV, residual variability.

**Table 4:** Components of simulation dataset for each experiment.

No. of Experiment	Scenario	Contents	Setting Value				
1st	1	WSV (%)	10	20	30	40	50
		IIV (%)	0				
		No. of subjects	6→12→18→24→30	6→12→18→24→30	6→12→18→24→30	6→12→18→24→30	6→12→18→24→30
2nd	1	WSV (%)	10				
		IIV (%)	10	20	30	40	50
		No. of subjects	6→12→18→24→30	6→12→18→24→30	6→12→18→24→30	6→12→18→24→30	6→12→18→24→30
	2	WSV (%)	20				
		IIV (%)	10	20	30	40	50
		No. of subjects	6→12→18→24→30	6→12→18→24→30	6→12→18→24→30	6→12→18→24→30	6→12→18→24→30
	3	WSV (%)	30				
		IIV (%)	10	20	30	40	50
		No. of subjects	6→12→18→24→30	6→12→18→24→30	6→12→18→24→30	6→12→18→24→30	6→12→18→24→30
	4	WSV (%)	40				
		IIV (%)	10	20	30	40	50
		No. of subjects	6→12→18→24→30	6→12→18→24→30	6→12→18→24→30	6→12→18→24→30	6→12→18→24→30
	5	WSV (%)	50				
		IIV (%)	10	20	30	40	50
		No. of subjects	6→12→18→24→30	6→12→18→24→30	6→12→18→24→30	6→12→18→24→30	6→12→18→24→30

Abbreviation: IIV, interindividual variability; WSV, within-subject variability.

## Data Availability

The data presented in this study are available upon request.

## Supplementary Materials

The following are available online at https://www.mdpi.com/1424-8247/14/2/114/s1. Figure S1: The proportion of predictive success for scenarios of the first experiment. Figure S2: The proportion of predictive success for scenarios of the second experiment. Table S1: Tabulated summary for results of the comparison, with and without covariance between omegas. Text S1: Sample R code for generating simulation dataset. Text S2: C++ and R script codes when using the mrgsolve R package. Text S3: NONMEM PK model code.
