# Supplementary material for: Population Pharmacokinetic Method to Predict Within-Subject Variability Using Single-Period Clinical Data
Source: Pharmaceuticals (Basel). 2021 Feb 3;14(2):114. doi: 10.3390/ph14020114 (PMC7913178; doi:10.3390/ph14020114)
Supplement: Supplementary file 1 [file pharmaceuticals-14-00114-s001.pdf]

Supplemental Material

Population pharmacokinetic method to predict within-subject variability using single-period clinical data

Won-ho Kang [1,¶], Jae-yeon Lee [1,2,¶], Jung-woo Chae [1], Kyeong-ryoon Lee [3], In-hwan Baek [4], Min-soo Kim [5], Hyun-moon Back [6], Sangkeun Jung [7], Craig H Shaffer [8], Rada Savic [8,*], Hwi-yeol Yun [1,*]

1 College of Pharmacy, Chungnam National University, Deajeon, Republic of Korea
2 Division of Convergence Technology New Drug Development Center, Osong Medical Innovation Foundation, Cheongju, Chungbuk, Republic of Korea
3 Laboratory Animal Resource Center, Korea Research Institute of Bioscience and Biotechnology, Ochang, Chungbuk, Republic of Korea
4 College of Pharmacy, Kyoungsung University, Busan, Republic of Korea
5 College of Pharmacy, Pusan National University, Busan, Republic of Korea
6 Department, Pharmaceutics, Ernest Mario School of Pharmacy, Rutgers, The State University of New Jersey, NJ, USA
7 Department of Computer Science and Engineering, Chungnam National University, Daejeon 34134, Republic of Korea
8 Department of Bioengineering and Therapeutic Sciences, School of Pharmacy, University of California San Francisco, CA, USA

¶ Those of authors were contributed equally for this work as first author
* Those of authors were contributed equally for this work as corresponding author

Contents

Figure S1 1

Figure S2 2

Table S1 3

Text S1 4

Text S2 6

Text S3 8

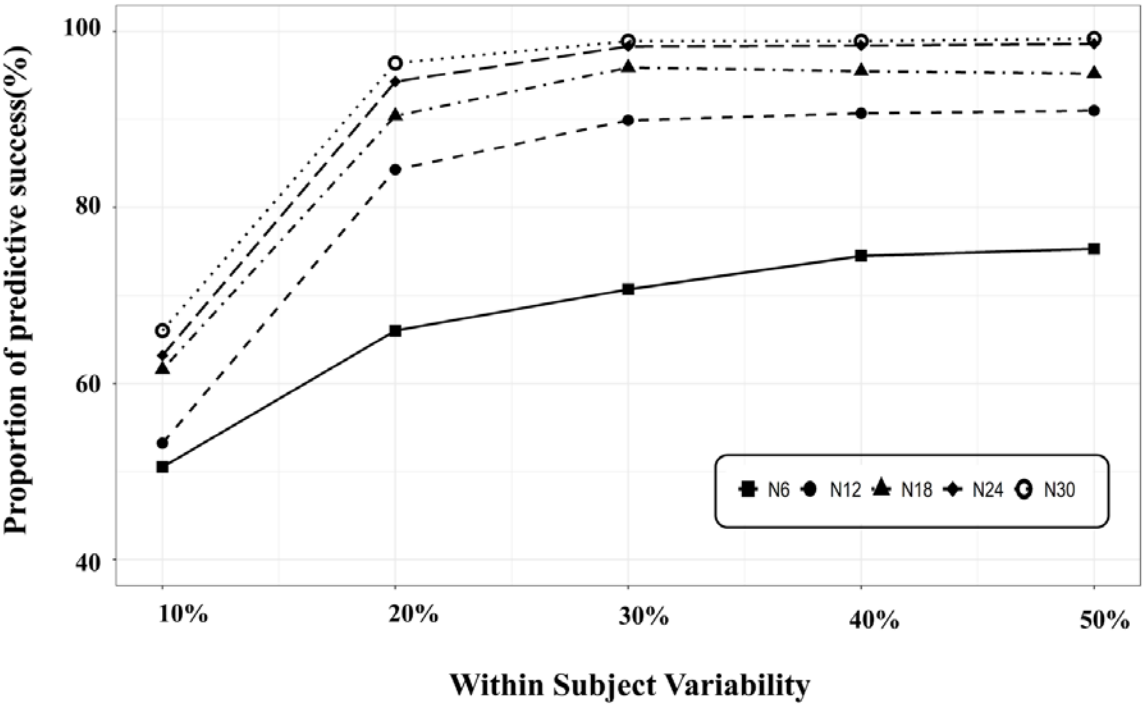

Figure S1. The proportion of predictive success for the first experiment.

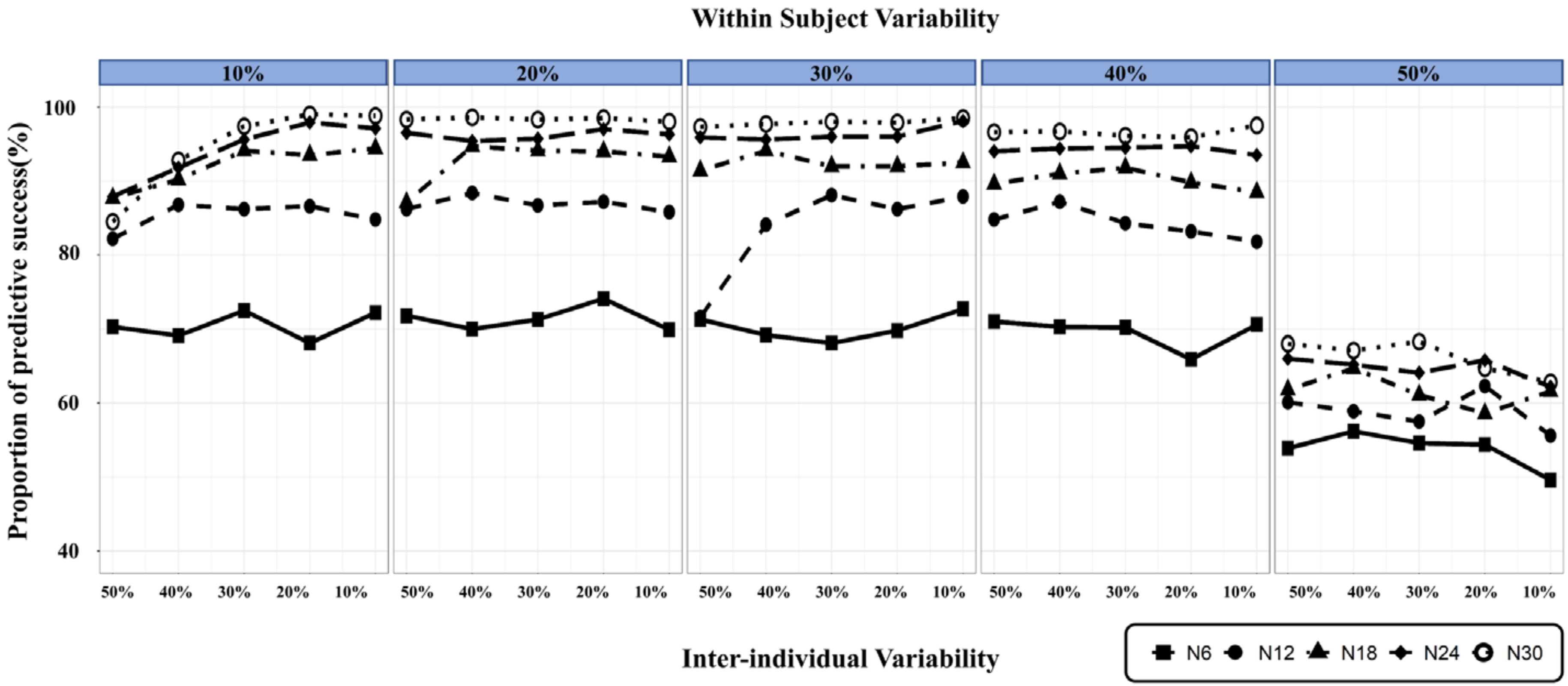

Figure S2. The proportion of predictive success for the second experiment.

Table S1. Tabulated summary for results of comparison with and without covariance between omegas

Evaluation scenario			Proportion of predictive success for RV(%)	
**WV(%)**	**IIV(%)**	**No. of subject**	**With Covariance between ω1 and ω2***	**Without Covariance between ω1 and ω2****
50	10	18	52	62
50	50	18	50	62
10	50	18	89	88

* Preform of NONMEM PK model for 100 simulation dataset generated by mrgsolve R package,
**Perform of NONMEM PK model for 1000 simulation dataset generated by basic R code. The ω1 and ω2 meant IIV on CL and Vd, respectively.

Text S1. Example as a R code for generating simulation dataset

```
## Individual PK parameters(IIV 10%)
data.sample <- 1:1000
start.number <- min(data.sample)
i <- start.number
  for (i in data.sample) {
     set.seed(seed[i])
     x <- rlnorm(100000, meanlog = 10, sdlog = 1)
     y <- rlnorm(100000, meanlog = 50, sdlog = 5)
     CL <- sample(log(x)[log(x)>0],12)      # Preventing for generating negative number at CL
     Vd <- sample(log(y)[log(y)>0],12)      # Preventing for generating negative number at Vd
     id <-seq(1:12)
     data <- data.frame(id,CL,Vd)
     write.table(data,paste("data",i,".csv",sep=""),sep=",",row.names = F)
}
## Calulation the concentration using parameters(CL, Vd)
total <- 1:1000
min.total <- min(total)
k <- min.total
for(k in total){
    one <- read.table(paste("data",k,".csv",sep=""),sep = ",",header=TRUE)
    kel <- one$CL/one$Vd
    one <- cbind(one,kel)
    number <- 1:12
    begin <- min(number)
    i <- begin
    for(i in number){
       special.ID <-DO[DO$ID==i,]
       timenumber <- 1:12
       time.start <- min(timenumber)
       j <- time.start
       for(j in timenumber){
          #iv PK equation(WV = 0.3)
          conco <- (100*exp(-one$kel[i]*special.ID$TIME[j])/(one$Vd[i]))
          eps <- rnorm(12, mean = 0, sd = 0.3)
          eps.sample <- sample(eps,1)
     conco2 <- conco*(1+eps.sample)      ##Proportional model
# Preventing for generating negative number at plasma concentration
          while(conco2<0){
             eps <- rnorm(12, mean = 0, sd = 0.3)
             eps.sample <- sample(eps,1)
             conco2 <- conco*(1+eps.sample)      ##Proportional model
          }
          if(j==time.start)
             DV <- conco2
          else DV <-rbind(DV,conco2)
       }
       real <- cbind(special.ID, DV)
       if(i==begin)
          set <- real
```

```
        else set <-rbind(set, real)
   }
write.table(set, paste("CONC",k,".csv",sep = ""),sep = ",", row.names = FALSE)}
```

Text S2. C++ and R script code when used mrgsolve R package

```
## Example of CPP file
$PROB
- 1 COMP iv PK model
- Random effect : yes
$PARAM
TVCL = 10
TVV = 50
$CMT
CENT
$MAIN
double CL=TVCL*exp(ETA(1));
double V=TVV*exp(ETA(2));
$ODE
dxdt_CENT = -(CL/V)*CENT;
$OMEGA >> annotated=TRUE, block = TRUE
  ECL: ETA on Clearance
  EV: ETA on Volume
0.25
0.0625 0.25
$SIGMA
0.25
$TABLE
double IPRED = CENT/V;
double DV = IPRED*(1+EPS(1));
$CAPTURE
DV IPRED
```

```
## Example of R script file
# problem : WV0.5_IIV0.5_omegacovariance0.5
# subject NO. = 18
# load packages
library(mrgsolve)
library(tidyverse)
library(dplyr)
# giving seed number
set.seed(20191203)
# loading cpp file
mod<-mread("WV0.5_IIV0.5_COV0.5","C:/Users/Wonho
Kang/Desktop/TEST_OMBLOCK/WV0.5_IIV0.5_COV0.5/N18")
total <- 1:100
min.total <- min(total)
i <- min.total
for(i in total){
  nn=18
  idata<-tibble(ID=seq(nn))
  tmptolerance = -1
  tmpcount = 0
  while (tmptolerance < 0){
  tmpcount = tmpcount +1
```

```
    df<-mod %>%
    ev(amt=100) %>%
    idata_set(idata) %>%
    mrgsim(end=24, delta=0.1) %>%
filter(time==0|time==0.083|time==0.167|time==0.333|time==0.5|time==1|time==2|time==4|time==6|ti
me==8|time==12|time==24 )
    tmptolerance = min(df$DV)
    print(tmpcount)
    }
    #add MDV
    df<-df %>% mutate(MDV=0)
    df$MDV[which(df$DV==0)]<-1
    #add AMT
    df<-df %>% mutate(AMT=0)
    df$AMT[which(df$MDV==1)]<-100
    #rename TIME
    df<-df %>% rename(TIME=time)
    df<-df %>% arrange(ID,TIME)
    df<-df[c("ID","TIME","DV","MDV","AMT")]
    names(df) <- c("#ID","TIME","DV","MDV","AMT")
    write.table(df,paste("datafile",i,".csv",sep = ""),sep = ",",quote=FALSE,row.names=FALSE)
  }
```

Text S3. NONMEM PK Model code

```
$PROBLEM Simulation dataset2_IIV50%_WV30%_N18
$INPUT ID TIME DV MDV AMT
$DATA datafile1.csv IGNORE=@
$SUBROUTINES ADVAN6 TOL=8
$MODEL
 COMP (CENT, DEFDOSE, DEFOBS)
$PK
  CL =   THETA(1) * EXP(ETA(1))
  V   =   THETA(2) * EXP(ETA(2))
  KEL = CL/V
  S1=V
$DES
  DADT(1) = -KEL*A(1)
$ERROR
  IPRED = F
  DEL=0
  IF(IPRED.EQ.0) DEL=1
  W=IPRED+DEL
  IRES = DV-IPRED
  IWRES = IRES/W
  Y = F+W*EPS(1)
$THETA
(0, 10) ; CL
(0, 50) ; V
$OMEGA
 0.25
 0.25
$SIGMA
 0.09
$EST METHOD=1 MAXEVAL=9999 NOABORT INTER PRINT=5 NSIG=2 SIGL=8
$TABLE ID TIME DV MDV AMT IPRED IWRES CWRES ONEHEADER NOPRINT FILE = sdtab1
$TABLE ID ETA1 ETA2 ONEHEADER NOPRINT FILE = patab1
```
